# Supplementary material for: Temporal and spatial differences in the post-breeding behaviour of a ubiquitous Southern Hemisphere seabird, the common diving petrel
Source: R Soc Open Sci. 2020 Nov 18;7(11):200670. doi: 10.1098/rsos.200670 (PMC7735348; doi:10.1098/rsos.200670)
Supplement: Supplementary Figures and Table [file rsos200670supp1.docx]

**Supplementary data**

**Temporal and spatial differences in the post-breeding behaviour of a ubiquitous Southern Hemisphere seabird, the common diving petrel**

Aymeric Fromant^a,b^, Charles-André Bost^b^, Paco Bustamante^c,d^, Alice Carravieri^c^, Yves Cherel^b^, Karine Delord^b^, Yonina H Eizenberg^a^, Colin M Miskelly^e^, John P.Y. Arnould^a^

^a^ School of Life and Environmental Sciences, Deakin University, 221 Burwood Hwy, Burwood, VIC 3125, Australia

^b^Centre d’Etudes Biologiques de Chizé (CEBC), UMR 7372 du CNRS - La Rochelle Université, 79360 Villiers en Bois, France

^c^Littoral Environnement et Sociétés (LIENSs), UMR 7266 CNRS - La Rochelle Université, 2 rue Olympe de Gouges, 17000 La Rochelle, France

^d^Institut Universitaire de France (IUF), 1 rue Descartes 75005 Paris, France

^e^Museum of New Zealand Te Papa Tongarewa, PO Box 467, Wellington 6140, New Zealand


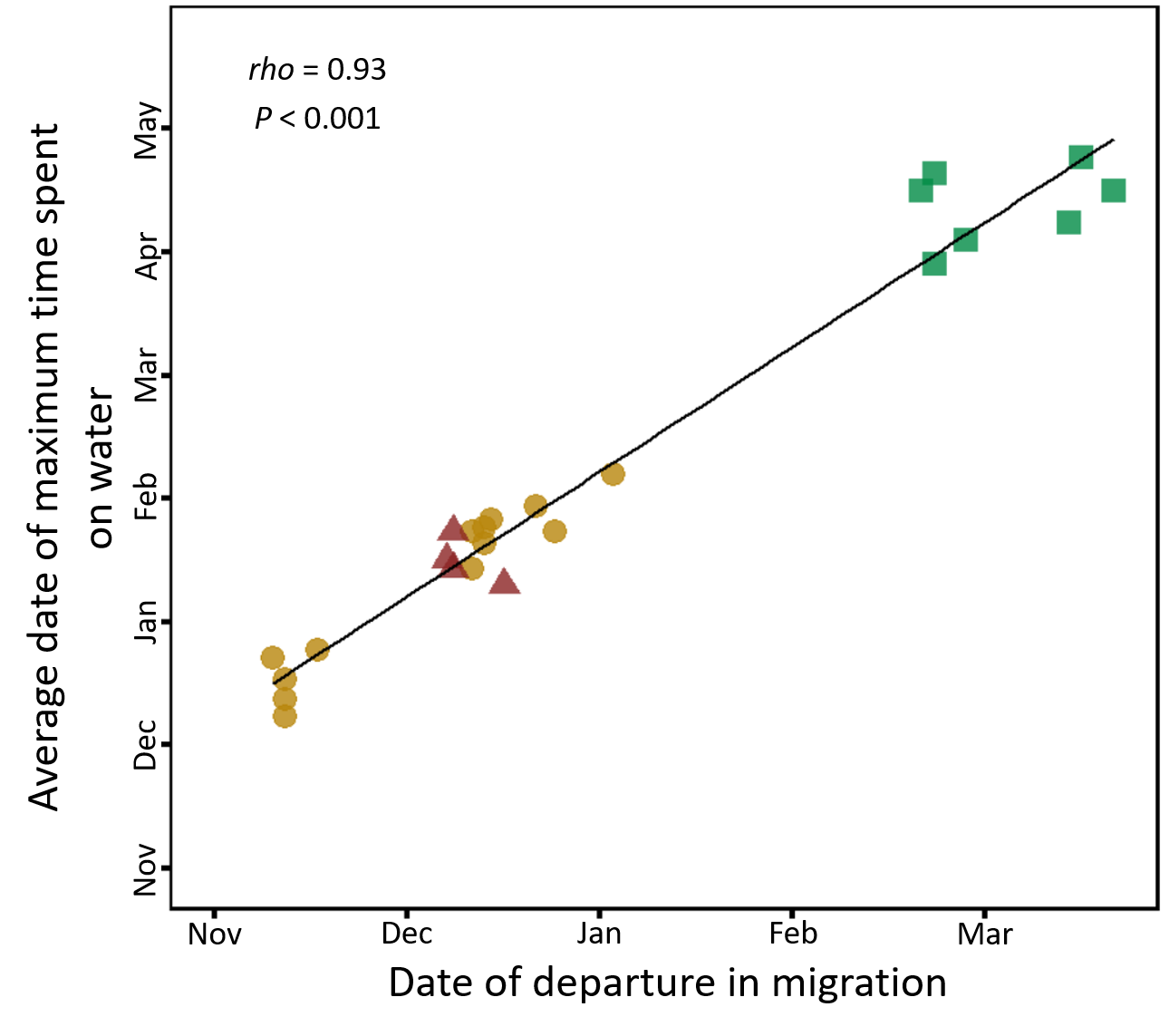


**Supplementary Figure S1**: Correlation between departure date and the average date of maximum time spent on the water (mean moult period) for common diving petrels from Kerguelen (green scares, 2017-18, N = 7), Kanowna (orange dots, 2017-18 and 2018-19, N =13), and Mana Islands (red triangle, 2017-18, N = 4). The Mean moult date was identified as the peak of time spend on the water (> 90% time per day sitting on water). Birds were equipped with miniaturized saltwater immersion geolocators.


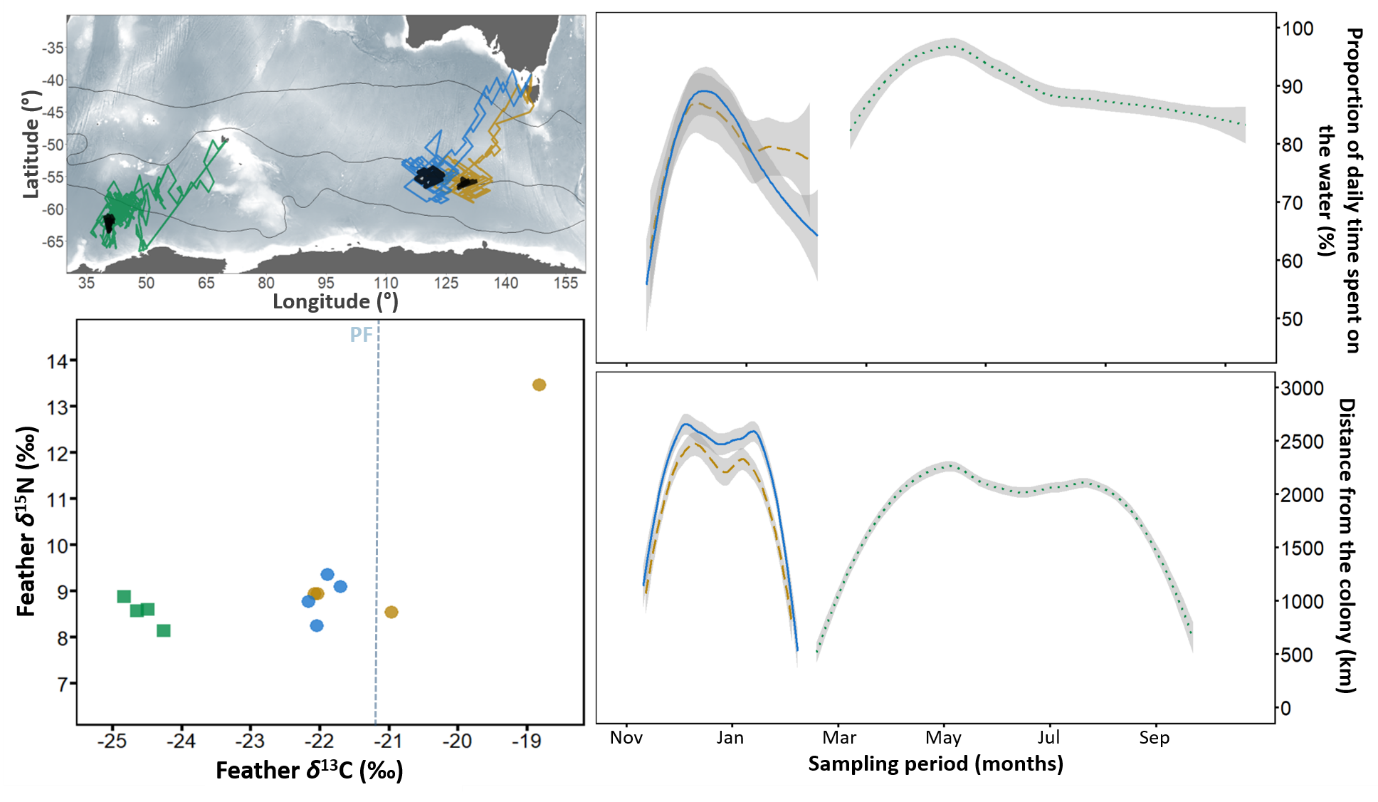


**Supplementary Figure S2**: Example of inter-population and intra-population variation of migratory behaviour during the post-breeding migration of common diving petrels from Kerguelen (1 individual in green) and Kanowna (2 individuals in blue and yellow). Lower left: stable carbon and nitrogen isotope values of body feathers (four per individual). The dotted line corresponds to the feather *δ*^13^C estimation of the Polar Front (PF) (Jaeger et al. 2010); Upper left: individual track derived from geolocator data. For each track, the estimated moult period of flight feathers is highlighted in black; Upper right: daily time spent on the water for each individual; Lower Right: distance from the colony for each individual. Data for upper and lower right panels were fitted with a generalized additive mixed models (GAMMs). The shaded areas along the curves represent the 95% confidence interval.

**
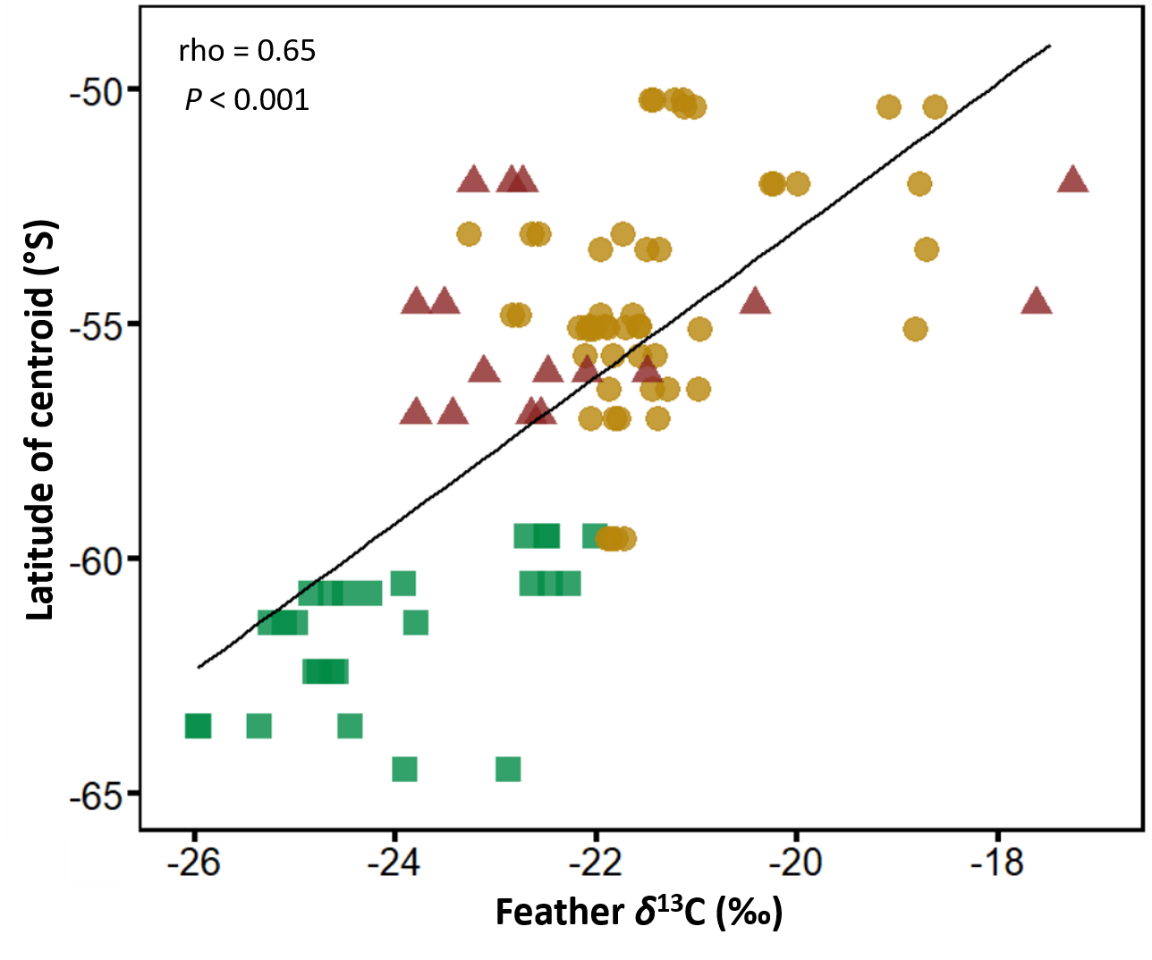
**

**Supplementary Figure S3**: Correlation between stable carbon isotopic values (δ^13^C) of body feathers (four per individuals) and the latitude of centroid of the 50% kernel utilization distribution (core area) for common diving petrels from Kerguelen (green squares; 2017-18, N = 7), Kanowna (orange dots; 2017-18 and 2018-19, N =13), and Mana Islands (red triangles; 2017-18, N = 4).

**
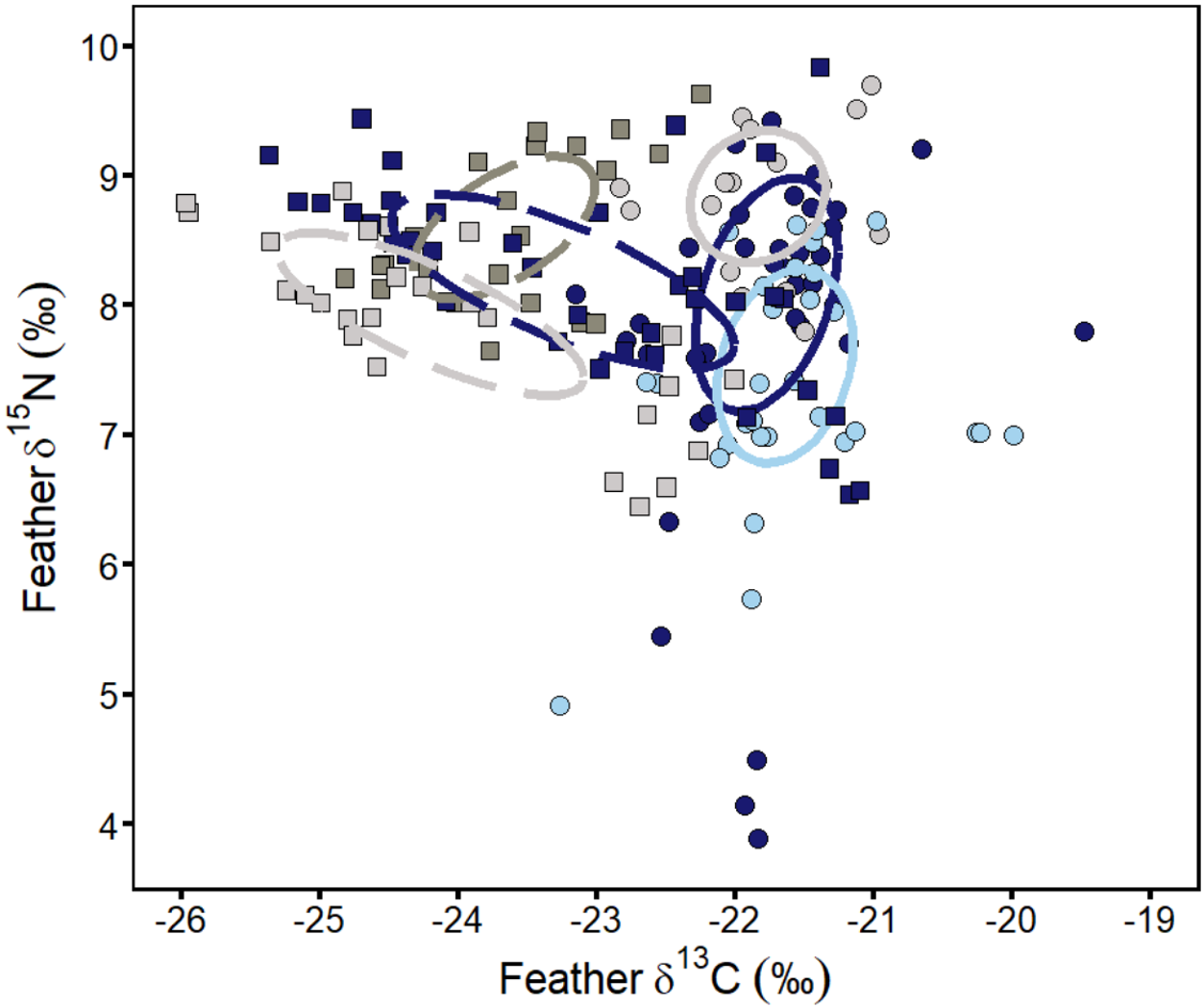
**

**Supplementary Figure S4**: Niche overlap of δ^13^C and δ^15^N values in body feathers (4 per individuals) of common diving petrel from Kerguelen (dashed line) and Kanowna (solid line). Dark grey = 2015-16 (Kerguelen only, N = 6), blue navy = 2016-17 (Kerguelen N = 10, Kanowna N = 12), grey = 2017-18 (Kerguelen N = 7, Kanowna N = 5), light blue = 2018-19 (Kanowna only, N = 8). Solid lines represent the standard ellipses corrected for sample size (SEAc; 40% probability of containing a subsequently sampled datum regardless of sample size; Jackson et al. 2011).

**
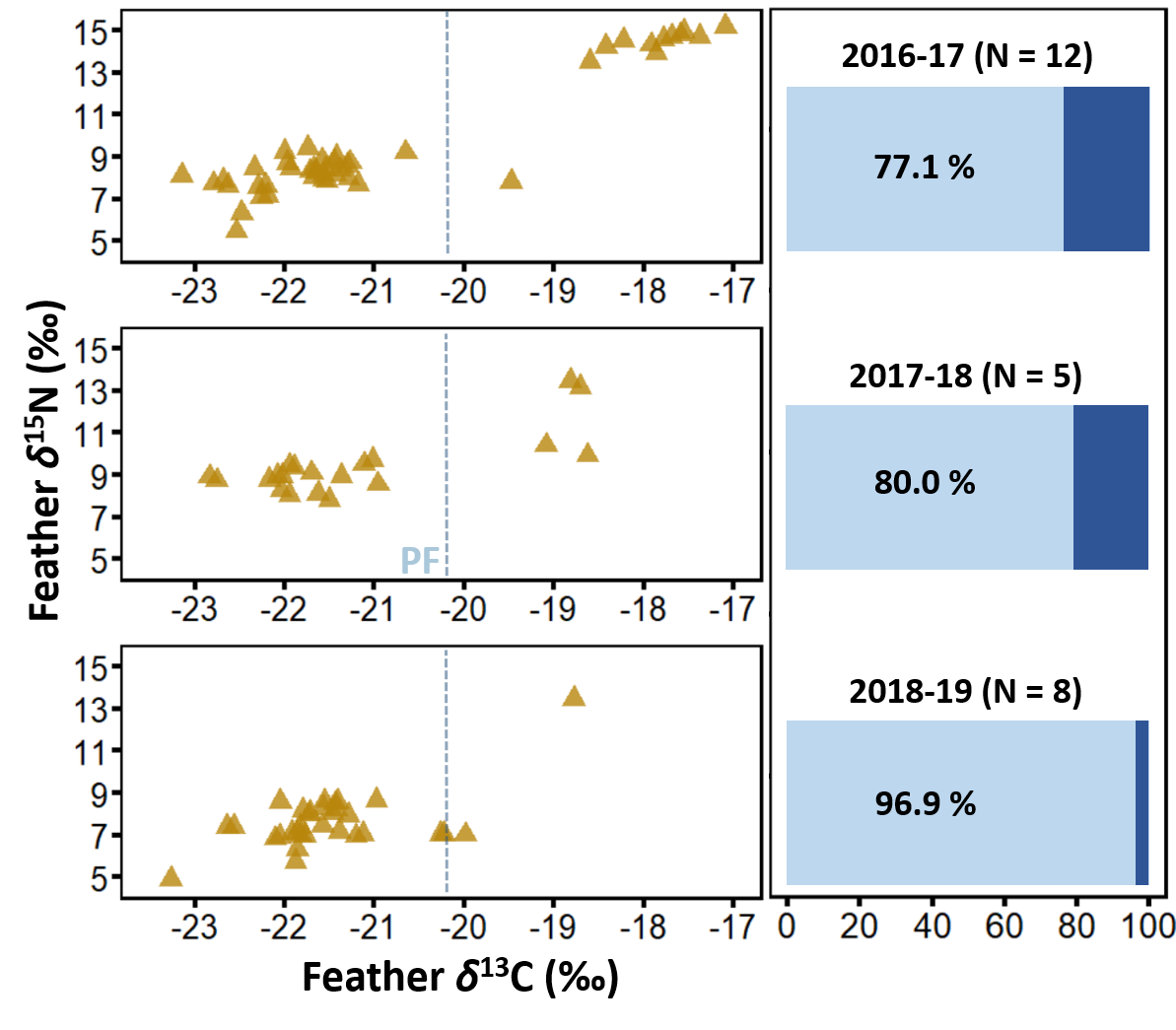
**

**Supplementary Figure S5**: Inter-annual variation of isotopic values in body feathers of common diving petrel from Kanowna (four feathers per individual). The right panel presents the proportion of feathers moulted in Antarctic waters (percentage; light blue) or in sub-tropical waters (dark blue). The dotted line corresponds to the feather *δ*^13^C estimation of the Polar Front (PF) (Jaeger et al. 2010). Departure in post-breeding migration was unknown in 2017, but was, early November and early December in 2018 and 2019, respectively (Eizenberg 2019).

**Supplementary Table 1: Results of the GLM models explaining trip parameters (duration of post-breeding period, departure date, return date, maximum range, and total distance travelled) for common diving petrels Kerguelen (2015-16, n = 5; 2017-18 = 7), Kanowna (2017-18, n = 5; 2018-19, n =8) and Mana Islands (2017-18, n = 4).**

Explanatory variables, deviance explained, Akaike Information Criterion (AIC) and Delta AIC (difference in AIC with the best model) are given for each model. Models are ranked according to their respective AIC value and significant variables in each model are highlighted in bold.

|  | Model | Deviance | AIC | Delta AIC |
| --- | --- | --- | --- | --- |
| Total duration of post-breeding period | DURATION ~ **POPULATION** + YEAR | 91.1 % | 216.6 | 0.0 |
|  | DURATION ~ POPULATION + YEAR + SEX | 91.3 % | 219.9 | 3.4 |
|  | DURATION ~ POPULATION | 84.6 % | 226.2 | 9.6 |
|  | DURATION ~ 1 | 0.0 % | 268.9 | 52.4 |
|  | DURATION ~ YEAR | 11.3 % | 269.9 | 53.4 |
|  | DURATION ~ SEX | 1.1 % | 272.6 | 56.1 |
| Departure date | DEPARTURE ~ **POPULATION** + **YEAR** | 92.1 % | 234.0 | 0.0 |
|  | DEPARTURE ~ POPULATION + YEAR + SEX | 92.3 % | 237.1 | 3.1 |
|  | DEPARTURE ~ POPULATION | 85.5 % | 246.9 | 12.9 |
|  | DEPARTURE ~ YEAR | 27 % | 292.1 | 58.1 |
|  | DEPARTURE ~ 1 | 0.0 % | 296.9 | 62.9 |
|  | DEPARTURE ~ SEX | 3.7 % | 299.8 | 65.8 |
| Return date | RETURN ~ **POPULATION** + YEAR | 98.3 % | 206.5 | 0.0 |
|  | RETURN ~ POPULATION + YEAR + SEX | 98.5 % | 207.2 | 0.7 |
|  | RETURN ~ POPULATION | 92.3 % | 239.7 | 33.1 |
|  | RETURN ~ YEAR | 15.4 % | 299.7 | 93.1 |
|  | RETURN ~ 1 | 0.0 % | 299.8 | 93.2 |
|  | RETURN ~ SEX | 3.1 % | 303.1 | 96.5 |
| Maximum range | RANGE ~ **POPULATION** | 38.4 % | 442.0 | 0.0 |
|  | RANGE ~ POPULATION + YEAR | 44.8 % | 442.8 | 0.8 |
|  | RANGE ~ POPULATION + YEAR + SEX | 45.3 % | 446.6 | 4.5 |
|  | RANGE ~ YEAR | 16.8 % | 450.7 | 8.7 |
|  | RANGE ~ 1 | 0.0 % | 452.1 | 10.0 |
|  | RANGE ~ SEX | 2.3 % | 455.4 | 13.3 |
| Total distance | DISTANCE ~ **POPULATION** + SEX | 52.6 % | 516.7 | 0.0 |
|  | DISTANCE ~ POPULATION | 40.8 % | 518.2 | 1.6 |
|  | DISTANCE ~ POPULATION + YEAR + SEX | 53.1 % | 520.4 | 3.7 |
|  | DISTANCE ~ 1 | 0.0 % | 527.4 | 10.7 |
|  | DISTANCE ~ YEAR | 10.9 % | 528.5 | 11.8 |
|  | DISTANCE ~ SEX | 4.0 % | 530.3 | 13.6 |

**Reference:**

Eizenberg YH (2019) Breeding biology of sympatric fairy prions (*Pachyptila turtur*) and common diving petrels (*Pelecanoides urinatrix*) in northern Bass Strait. Deakin University, Melbourne

Jackson AL, Inger R, Parnell AC, Bearhop S (2011) Comparing isotopic niche widths among and within com munities: SIBER Stable Isotope Bayesian Ellipses in R. J Anim Ecol 80:595–602

Jaeger A, Lecomte VJ, Weimerskirch H, Richard P, Cherel Y (2010) Seabird satellite tracking validates the use of latitudinal isoscapes to depict predators’ foraging areas in the Southern Ocean. Rapid Comm Mass Spectrom 24:3456–3460. doi:10.1002/rcm.4792 PMID: 21072802
